# Effect of temporal resolution on calcium scoring: insights from photon-counting detector CT

**DOI:** 10.1007/s10554-024-03070-6

**Published:** 2024-02-23

**Authors:** Thomas Sartoretti, Victor Mergen, Amina Dzaferi, Thomas Allmendinger, Robert Manka, Hatem Alkadhi, Matthias Eberhard

**Affiliations:** 1 Diagnostic and Interventional Radiology, University Hospital Zurich, University of Zurich, Raemistrasse 100, 8091, Zurich, Switzerland; 2 Siemens Healthcare GmbH, Computed Tomography, Forchheim, Germany; 3 Department of Cardiology, University Heart Center, University Hospital Zurich, University of Zurich, Zurich, Switzerland; 4 Radiology, Spital Interlaken, Spitäler fmi AG, Unterseen, Switzerland

**Keywords:** Computed tomography, Photon-counting detector, Calcium scoring, Temporal resolution

## Abstract

To intra-individually investigate the variation of coronary artery calcium (CAC), aortic valve calcium (AVC), and mitral annular calcium (MAC) scores and the presence of blur artifacts as a function of temporal resolution in patients undergoing non-contrast cardiac CT on a dual-source photon counting detector (PCD) CT. This retrospective, IRB-approved study included 70 patients (30 women, 40 men, mean age 78 ± 9 years) who underwent ECG-gated cardiac non-contrast CT with PCD-CT (gantry rotation time 0.25 s) prior to transcatheter aortic valve replacement. Each scan was reconstructed at a temporal resolution of 66 ms using the dual-source information and at 125 ms using the single-source information. Average heart rate and heart rate variability were calculated from the recorded ECG. CAC, AVC, and MAC were quantified according to the Agatston method on images with both temporal resolutions. Two readers assessed blur artifacts using a 4-point visual grading scale. The influence of average heart rate and heart rate variability on calcium quantification and blur artifacts of the respective structures were analyzed by linear regression analysis. Mean heart rate and heart rate variability during data acquisition were 76 ± 17 beats per minute (bpm) and 4 ± 6 bpm, respectively. CAC scores were smaller on 66 ms (median, 511; interquartile range, 220–978) than on 125 ms reconstructions (538; 203–1050, *p* < 0.001). Median AVC scores [2809 (2009–3952) versus 3177 (2158–4273)] and median MAC scores [226 (0-1284) versus 251 (0-1574)] were also significantly smaller on 66ms than on 125ms reconstructions (*p* < 0.001). Reclassification of CAC and AVC risk categories occurred in 4% and 11% of cases, respectively, whereby the risk category was always overestimated on 125ms reconstructions. Image blur artifacts were significantly less on 66ms as opposed to 125 ms reconstructions (*p* < 0.001). Intra-individual analyses indicate that temporal resolution significantly impacts on calcium scoring with cardiac CT, with CAC, MAC, and AVC being overestimated at lower temporal resolution because of increased motion artifacts eventually leading to an overestimation of patient risk.

**Supplementary Information:**

The online version contains supplementary material available at 10.1007/s10554-024-03070-6.

## Introduction

Computed tomography (CT) is the modality of choice for the assessment and quantification of cardiac calcifications. Coronary artery calcium (CAC) scoring by computed tomography (CT) improves individual risk stratification for future cardiovascular events [1–3]. Aortic valve calcium (AVC) and mitral annular calcium (MAC) scores have shown to be valuable means for grading aortic stenosis severity [4, 5] and for predicting conduction system abnormalities after transcatheter aortic valve replacement (TAVR) [6, 7].

However, the accuracy and reliability of CT-based calcium quantification is affected by several factors, including the respective CT scanner [8, 9], slice thickness [10], the type and strength level of iterative reconstruction [11–13], and the cardiac phase [8, 14, 15]. Phantom studies indicated that another important factor impacting calcium quantification is coronary motion during data acquisition [15–17].

Depending on the heart rate and anatomical location, coronary arteries have very different velocities of motion during the cardiac cycle [18, 19]. The smaller the anatomical structures, such as calcified coronary atherosclerotic plaques, the greater the influence of motion on image blurring and loss of image sharpness [20]. This implies that a sufficiently high temporal resolution is crucial to accurately image small anatomical structures moving at high velocities. Consequently, in the past decades, manufacturers have continuously improved temporal resolution of CT scanners to achieve diagnostic image quality even at higher and irregular heart rates leading to a maximum temporal resolution of 66 ms for latest generation dual-source energy-integrating detector (EID) or the recently introduced dual-source photon-counting detector (PCD) CT systems [21], and 115 ms for single-source EID-CT systems [22]. To the best of our knowledge, the impact of temporal resolution of the scanner on calcium scoring in cardiac CT at an individual patient level has not been systematically evaluated yet.

Thus, the aim of this study was to intra-individually investigate the impact of temporal resolution on the accuracy of CAC, AVC and MAC scores in patients undergoing non-contrast cardiac CT.

## Materials and methods

### Patient sample

In this retrospective study, consecutive patients undergoing an electrocardiogram (ECG)-gated non-contrast scan as part of planning for TAVR on a first-generation dual-source PCD-CT between April 2022 and March 2023 were searched. Patients were included when they provided written consent for inclusion of their data in retrospective anonymized research and when raw data was available. The study had local ethics committee approval and was conducted in compliance with the declaration of Helsinki.

**Fig. 1:**
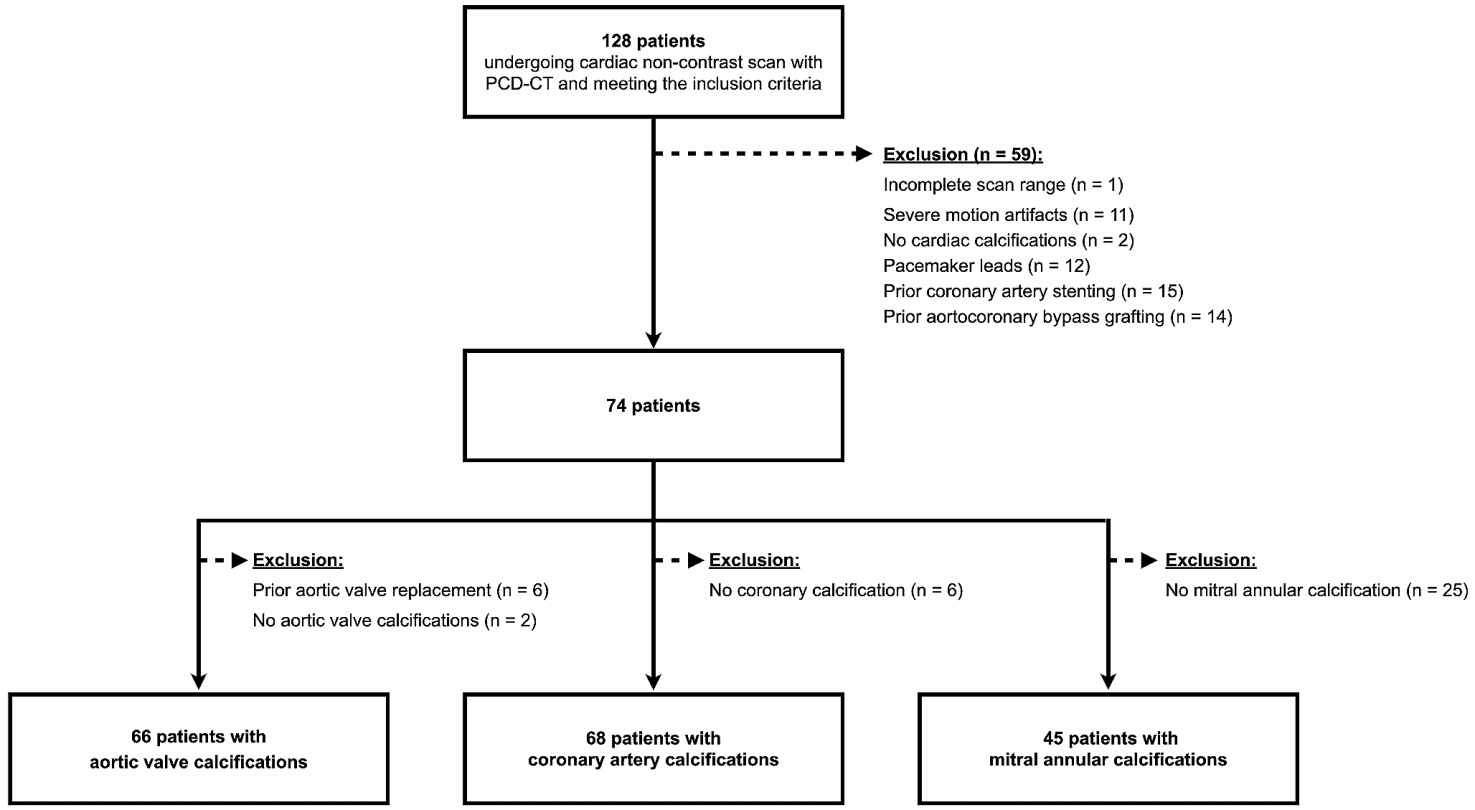
Flowchart detailing patient inclusion

One hundred twenty-eight patients who underwent a non-contrast cardiac scan with a PCD-CT and met the inclusion criteria were identified. 59 patients were excluded because of the following reasons: one scan was excluded because of an incomplete scan range not entirely including the heart, three patients had no cardiac calcifications, and 55 patients were excluded because of pacemaker leads, prior coronary artery stenting and prior aortocoronary bypass grafts as artifacts from foreign material affect image quality and hence, calcium quantification. A detailed flow chart is presented as Fig. 1.

### CT data acquisition protocol

All scans were performed on a dual-source PCD-CT system (NAEOTOM Alpha, version syngo CT VA50, Siemens Healthcare GmbH, Forchheim, Germany). Cardiac non-contrast scans were acquired in the dual-source prospective ECG-gated sequential mode at an absolute interval of 280 ms from the R wave applying a tube voltage of 120 kV and an image quality level of 20. The image quality level automatically adapts the tube current-time product using automated tube current modulation. Gantry rotation time was 0.25 s.

After acquisition of the non-contrast scan, the scan protocol included the acquisition of a coronary CT angiography, a CT aortography, and a late enhancement scan. No β-blockers were administered for heart rate control.

### CT image reconstruction

Non-contrast scans were reconstructed using a dedicated reconstruction software (recon CT, Version 16.0.1.2, Siemens). First, data from both measurement systems (dual-source mode) served to reconstruct the images. In the scanner, the two X-ray tubes are arranged in an angle of 95°. The measurement system uses data of slightly more than a quarter rotation in parallel geometry (after re-binning from fan beam geometry), which results in a temporal resolution of 66 ms at the iso-center for 0.25 s gantry rotation time [2]. The performance of a fictitious single source CT with the same geometry was simulated by using data from only one measurement system. Data from minimum half a rotation in parallel geometry is needed to reconstruct an image resulting in a temporal resolution of 125 ms at 0.25 s gantry rotation time. Both images reconstructed using dual-source and single source CT data had full image quality in terms of applied dose.

Images were reconstructed as virtual mono-energetic images at 70 keV without quantum iterative reconstruction (QIR), employing a slice thickness of 3 mm and an increment of 1.5 mm, and utilizing the Qr40 kernel [23, 24]. The matrix size was set to 512 × 512 pixels and the field-of-view was fixed at 240 × 240 mm^2^ for both reconstructions. Images with a temporal resolution of 66 ms served as the reference standard.

### Calcium scoring

One reader ([A.A.], medical student) quantified cardiac calcifications using a dedicated, commercially available, semi-automatic software (CaScoring, Syngo.via, Siemens) in accordance with the Agatston method. All measurements were checked by a second reader ([V.M.], in training with 4 years of experience in cardiovascular radiology). Calcium scores were reported for all coronaries, the left main coronary artery (LM), the left anterior descending coronary artery (LAD), the right coronary artery (RCA), and the circumflex coronary artery (CX). In addition, AVC scores and MAC scores were determined. In addition to Agatston scores (i.e. calcium scores), calcium mass and volume scores were also recorded.

CAC scores were classified into four risk categories using score boundaries of 0, 1–99, 100–299, and greater than 300, respectively [25]. For AVC scores, four risk categories served to stratify patients with respect to the likelihood of severe aortic stenosis: highly likely with a score greater than or equal to 1600 for women and greater than or equal to 3000 for men, likely with a score greater than or equal to 1200 for women and greater than or equal to 2000 for men, and unlikely with a score less than 800 for women and less than 1600 for men [26].

### Subjective image evaluation

Two radiologists with extensive experience in cardiovascular imaging ([H.A.], with 20 years of experience and [M.E.], with 10 years of experience) independently assessed images reconstructed with the two temporal resolutions in regard of motion blur affecting cardiac calcifications using a 4-point visual grading scale as follows: 1 – no motion artifacts with clear delineation of calcium, 2 – minor artifacts with mild blurring of calcifications, 3 – moderate artifacts with moderate blurring of calcifications, 4 – severe artifacts with doubling or discontinuity of calcium or severe artifacts.

### Statistical analysis

All analyses were performed in the R programming language (https://www.r-project.org/). To test for differences between the two reconstructions, Wilcoxon signed-rank tests or paired t-tests were used. Two-way intraclass correlation coefficients (ICC) optimized for agreement and Pearson correlation coefficients were used to quantify agreement in scores between reconstructions and/or between scores and demographic parameters. Additionally, Bland-Altman analyses were performed to quantify the relationship of scores between reconstructions. Wherever appropriate, p-values were adjusted for multiple comparisons using Bonferroni correction. Two-sided p-values < 0.05 were considered significant.

## Results

**Fig. 2:**
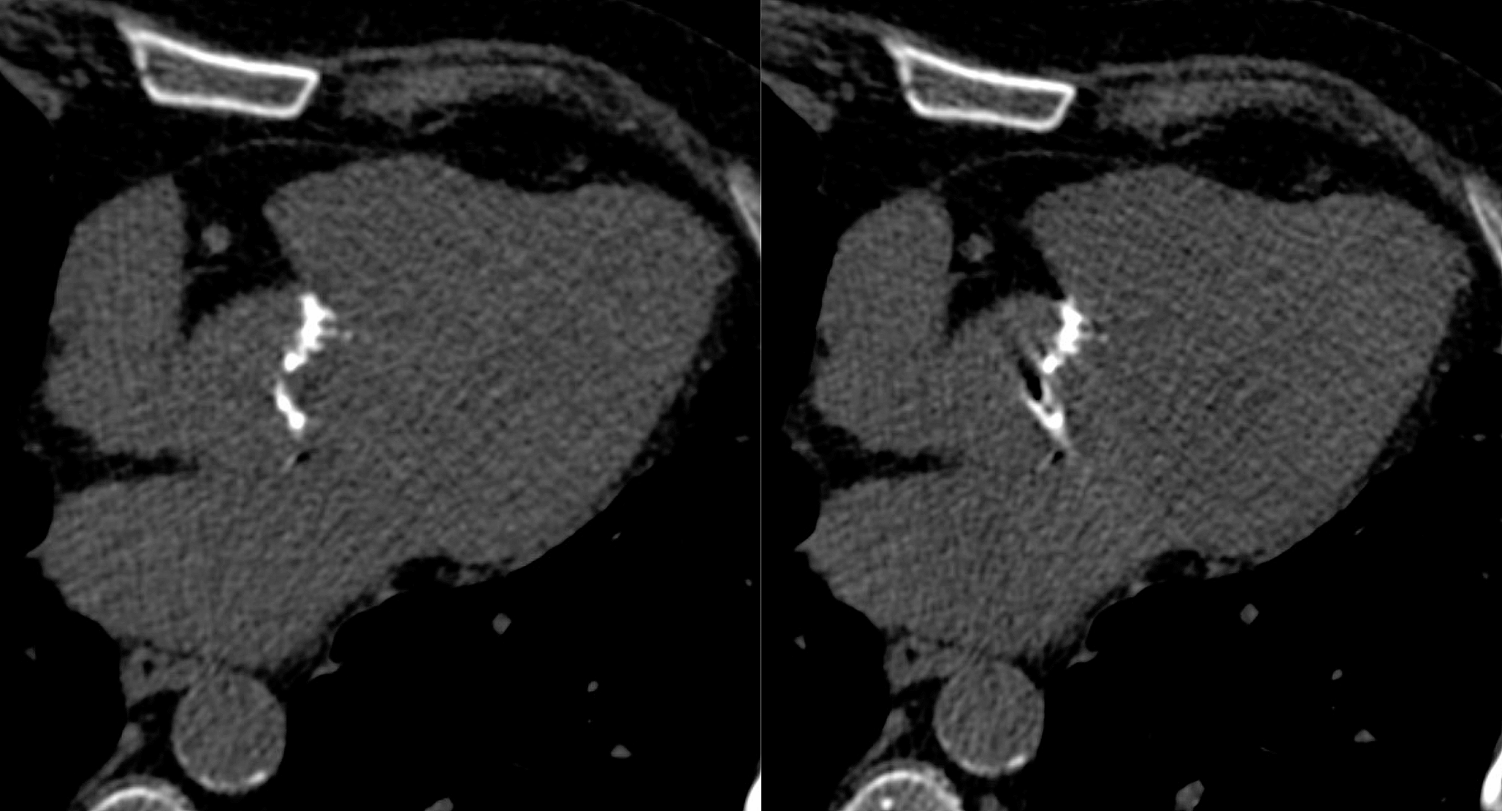
ECG-gated non-contrast CT images in a 65-year-old male patient with severe aortic stenosis and extensive aortic valve calcifications. Note the sharp delineation of aortic valve calcifications on 66 ms reconstructions (**A**) and the motion artifacts on 125 ms reconstructions (**B**). Aortic valve calcification scores were 3209 and 3839 on 66 ms and 125 ms reconstructions, respectively

**Fig. 3:**
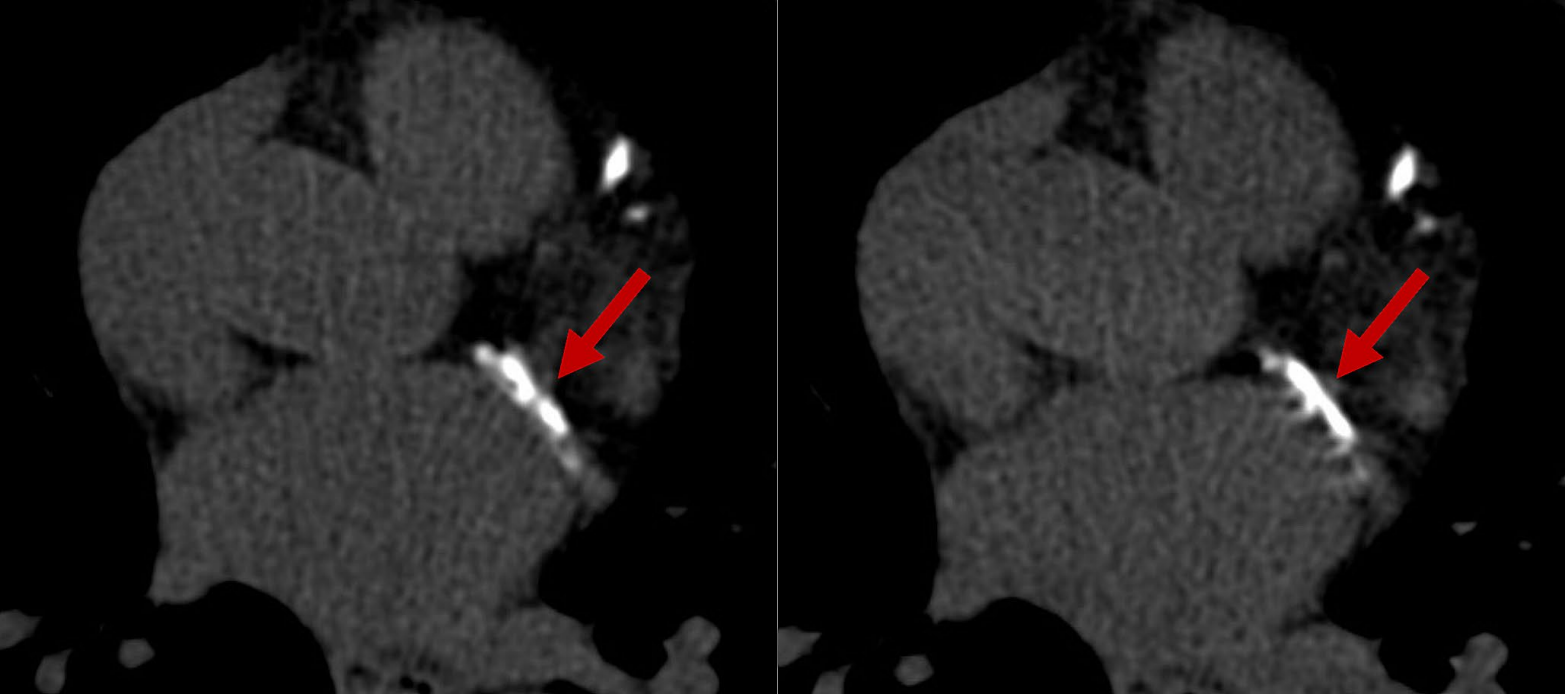
ECG-gated non-contrast CT images in a 65-year-old male patient with severe aortic stenosis and concomitant severe coronary artery calcifications. Sixty-six ms reconstructions show a sharp delineation of calcifications in the proximal circumflex artery (red arrow, **A**), whereas on 125 ms reconstructions severe motion artifacts can be observed (**B**)

A total of 70 patients (30 women, 40 men, mean age 78 ± 9 years, mean body mass index 27.1 ± 7.2 kg/m^2^) were included. Sixty-eight had coronary artery calcifications, 66 had aortic valve calcifications, and 45 had mitral annular calcifications. Patient demographics are shown as Table 1. Mean heart rate and heart rate variability during data acquisition were 76 ± 17 beats per minute (bpm) (range 51–136) and 4 ± 6 bpm (range 0–29), respectively. Figures 2 and 3 show representative images of patients undergoing cardiac non-contrast scans with PCD-CT reconstructed with temporal resolutions of 66 ms and 125 ms, respectively.

**Table 1:** Patient demographics

	All patients (*n* = 70)
*Sex*	
Women	30 (43%)
Men	40 (57%)
Age [years]	78 ± 9 (range, 58–93)
Body mass index [kg/m^2^]	26.6 ± 5.6 (range, 18.4–46.9)
Heart rate [bpm]	76 ± 17 (range, 51–136)
*Medical history*	
Hypertension	51/70 (73%)
Dyslipidemia	33/70 (47%)
Diabetes	27/70 (39%)
Smoking	24/70 (34%)
Chronic kidney disease (eGFR < 45 mL/min)	21/70 (30%)
*CT radiation dose*	
Volume CT dose index [mGy]	3.4 ± 1.7 (range, 1.6–12.1)
Dose length product [mGy⋅cm]	47.9 ± 20.2 (range, 21.4–149.0)
Size-specific dose estimates [mGy]	4.4 ± 1.5 (range, 2.9 ± 11.9)

Unless otherwise specified, data are mean ± standard deviation

bpm = beats per minute, eGFR = estimated glomerular filtration rate

### Calcium scoring

**Fig. 4:**
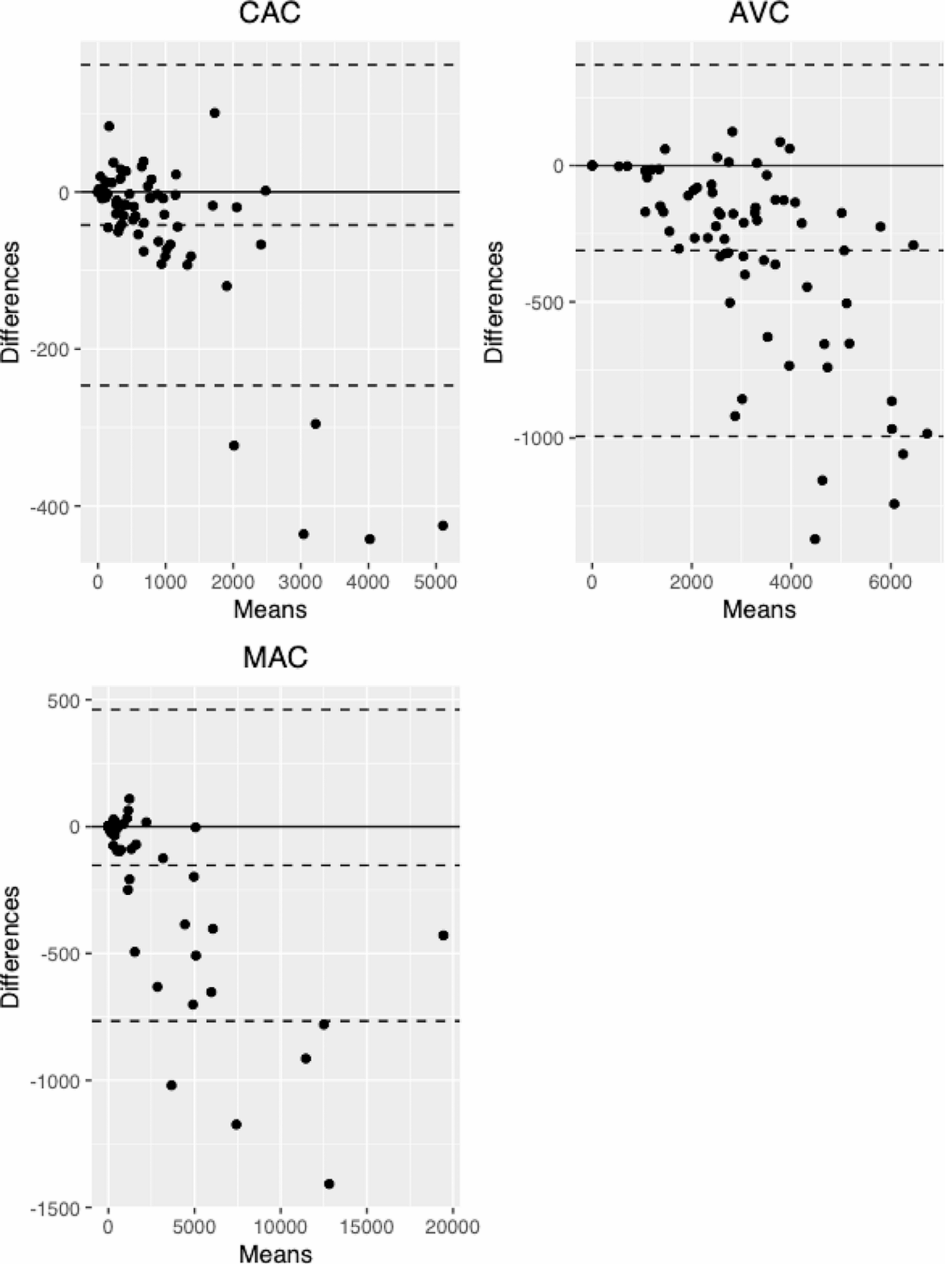
Bland-Altman plots for overall coronary artery calcium (CAC), aortic valve calcium (AVC) and mitral annular calcium (MAC)

A detailed overview of CAC, AVC and MAC scores stratified by temporal resolution is provided in Table 2. Bland-Altman plots are provided as Fig. 4. Additionally, calcium mass and volume scores for the coronaries, aortic valve and mitral valve, stratified by temporal resolution are provided in the **supplementary material**.

**Table 2:** Overview of coronary artery calcium (CAC), aortic valve calcium (AVC), and mitral annular calcium (MAC) scores stratified by temporal resolution. Scores are provided as median (interquartile range). P-values and ICC scores (with 95% confidence intervals) for groupwise comparisons (66ms vs. 125ms temporal resolution) are shown

	66 ms	125 ms	p-value	ICC	r	Difference (95% CI)	LoA (ULoA / LLoA)
Overall CAC	511 (220, 978)	538 (203, 1050)	< 0.001	0.993 (0.988, 0.996)	0.998	-42.2 (-67.1, -17.4)	162/-246.4
LM CAC	1.05 (0, 64.3)	1.85 (0, 64.6)	0.01	0.989 (0.982, 0.993)	0.996	-4 (-8.2, 0.1)	29.9/-38
LAD CAC	171 (64.6, 440)	187 (73.1, 450)	< 0.001	0.997 (0.995, 0.998)	0.998	-10.6 (-17.5, -3.7)	45.9/-67.1
CX CAC	67.6 (5.5, 212)	67.2 (5.22, 211)	**0.07**	0.982 (0.971, 0.989)	0.99	-5.5 (-16.3, 5.3)	83.6/-94.6
RCA CAC	121 (8.48, 385)	110 (4.45, 406)	0.004	0.987 (0.977, 0.992)	0.994	-22.1 (-37.9, -6.3)	107.8/-152
AVC	2809 (2009, 3952)	3177 (2158, 4273)	< 0.001	0.96 (0.785, 0.985)	0.986	-312.1 (-395, -229.1)	369.7/-993.9
MAC	226 (0, 1284)	251 (0, 1574)	< 0.001	0.995 (0.99, 0.998)	0.998	-152.5 (-227.1, -77.9)	460.8/-765.8

*Abbreviations* Cx, Circumflex coronary artery; ICC; intraclass correlation coefficients; LAD, left anterior descending; LM, left main coronary artery; LLoA, lower level of agreement; LoA, level of agreement; RCA, right coronary artery; ULoA, upper level of agreement

### Coronary calcifications

Median CAC scores were higher on 125 ms reconstructions than on 66 ms reconstructions both overall and on a per-vessel basis. Median overall CAC scores were 511 (interquartile range, 220–978) on 66 ms reconstructions and 538 (203–1050) on 125 ms reconstructions (*p* < 0.001), with a mean difference of 42 (limits of agreement − 162/246). Agreement as well as correlation of scores quantified on both reconstructions were high (ICC 0.998, *r* = 0.998).

38/70 (54%), 57/70 (81%), and 61/70 (87%) patients exhibited CAC in the LM, CX, and RCA vessels, respectively. For the LAD, 64/70 (91%) patients exhibited CAC on 66 ms reconstructions and 63/70 (90%) patients exhibited CAC on 125ms reconstructions. CAC scores quantified on the 66 ms and 125 ms reconstructions were significantly different for the LM, LAD, and RCA, respectively (all, *p* ≤ 0.01) while no significant differences were observed for the CX (*p* = 0.07). Agreement of scores quantified on both reconstructions was high for all segments (ICC ranging from 0.99 for the CX to 0.998 for the LAD). For the LAD, there was a significant impact of average heart rate on the absolute difference in calcium scores between both reconstructions (*r* = 0.3, *p* = 0.013) while for heart rate variability no significant relationship was found (*r* = 0.09, *p* = 0.46). For the other vessels, no significant impact of average heart rate or heart rate variability on the absolute difference in calcium scores between both reconstructions was found (*p* = 0.07 – *p* = 0.85).

**Fig. 5:**
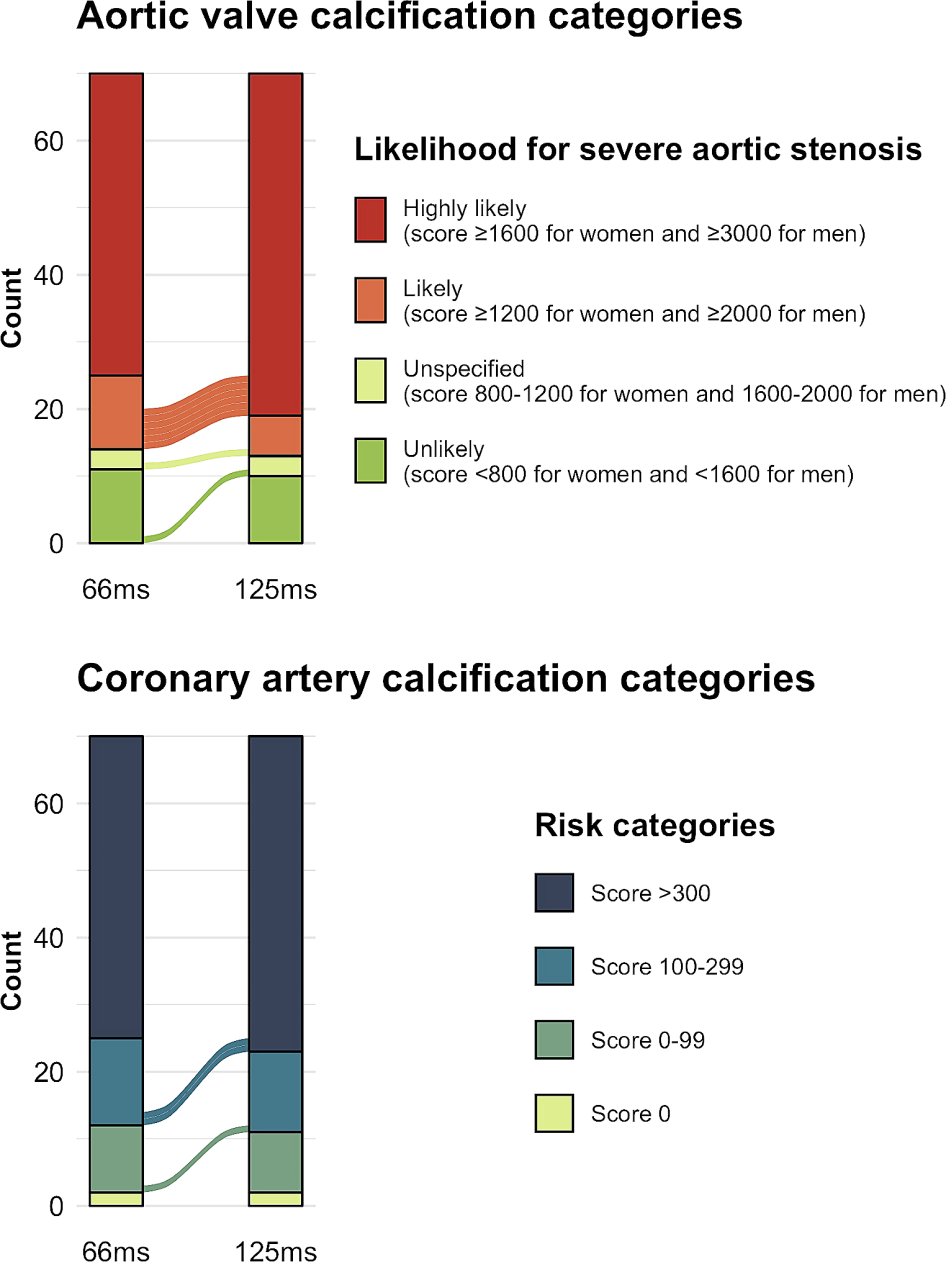
Barplots showing classification and reclassification rates of risk categories for AVC and CAC. Note the overestimation of categories on 125ms reconstructions both for AVC and CAC in 8 and 3 cases, respectively

Considering CAC risk categories, 3/70 (4%) patients were reclassified, whereby the risk category was overestimated by one category on 125 ms reconstructions as opposed to 66 ms reconstructions (Fig. 5).

### Aortic valve calcifications

Median AVC score was 2809 (2009–3952) on 66 ms reconstructions and 3177 (2158–4273) on 125 ms reconstructions (*p* < 0.001), with a mean difference of 312 (limits of agreement − 370/994) between both reconstructions. Agreement of AVC scores was high and correlation was strong (ICC 0.96, *r* = 0.99) between both reconstructions.

There was no significant correlation between average heart rate and the absolute difference in AVC scores between both reconstructions (*r* = 0.14, *p* = 0.26). However, there was a significant impact of heart rate variability on the absolute difference in AVC scores between both reconstructions (*r* = 0.27, *p* = 0.03).

Considering the likelihood for severe aortic stenosis categorization, 8/70 (11%) patients were reclassified, whereby the risk category was always overestimated on 125 ms reconstructions by one category (Fig. 5).

### Mitral annular calcifications

Median MAC score quantified was 226 (0-1284) on 66 ms reconstructions and 251 (0-1574) on 125 ms reconstructions. MAC scores were significantly different on both reconstructions (*p* < 0.001) and the mean difference of scores was 153 (limits of agreement − 461/766). Agreement of scores was high (ICC 0.995) and the correlation was excellent (*r* = 0.998). There was no significant correlation between average heart rate or heart rate variability and the absolute difference in MAC scores between both reconstructions (*r*=-0.1, *p* = 0.5 and *r* = 0.12, *p* = 0.45).

### Subjective image evaluation

**Fig. 6:**
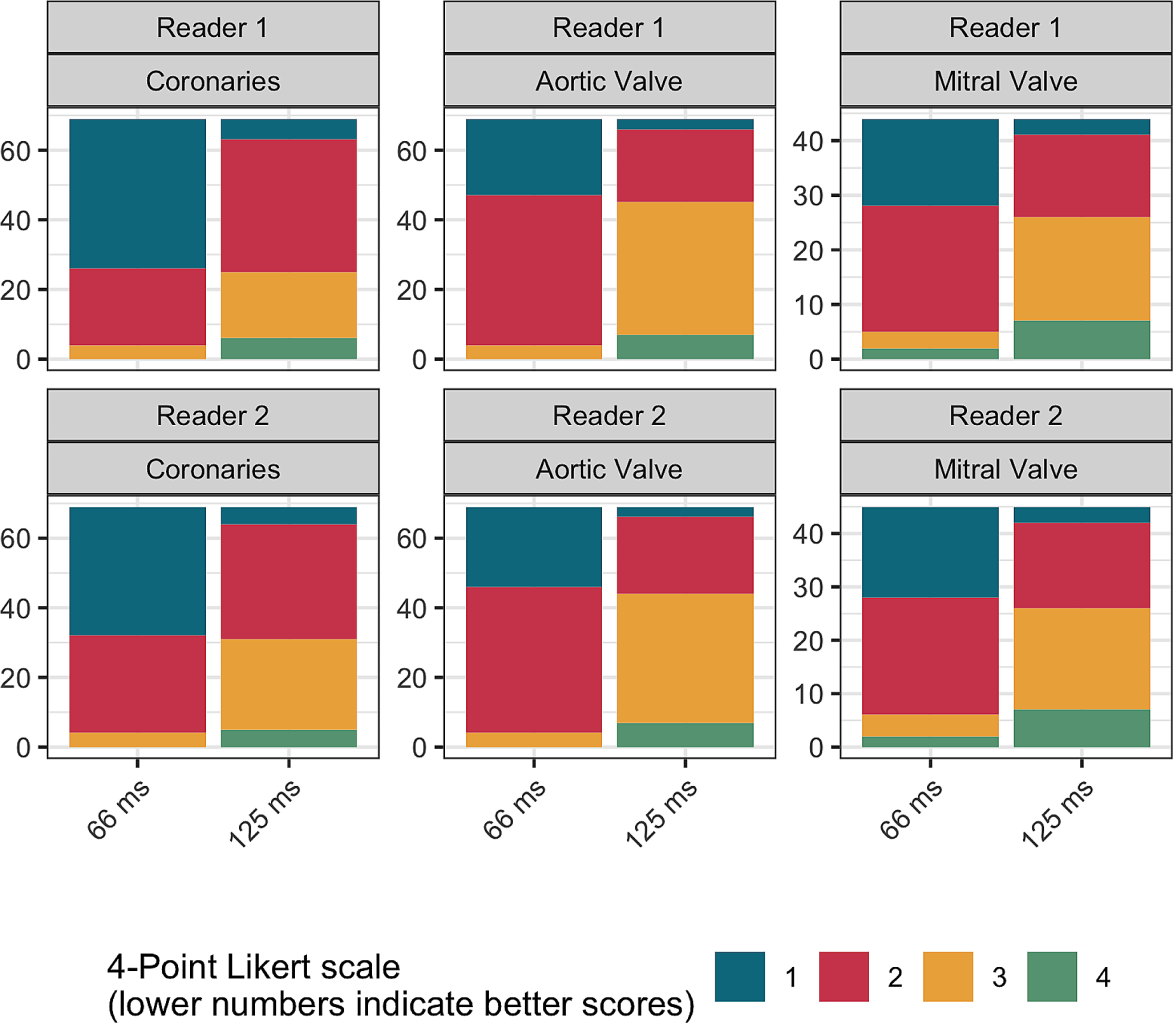
Barplots showing subjective image quality scores stratified by category, reader and reconstruction

Figure 6; Table 3 details the scores from subjective image evaluation. In brief, interreader agreement was high (ICC of 0.938) and the 66 ms reconstructions outperformed the 125 ms reconstructions in terms of artifact burden for CAC, AVC, and MAC evaluation, respectively, for both readers (*p* < 0.001). Specifically, for CAC, 66 ms reconstructions exhibited a median score of 1 while 125 ms reconstructions exhibited a median score of 2 for both readers (*p* < 0.001). Accordingly, for both readers the median score dropped from 2 to 3 from 66 ms to 125 ms reconstructions both for AVC and MAC (both, *p* < 0.001).

**Table 3:** Overview of scores from subjective image analysis. Scores are shown as median (interquartile range) and are stratified by reconstruction, reader, and type of calcification

	CAC	AVC	MAC
66ms	Reader 1: 1 (1,2) Reader 2: 1 (1,2)	Reader 1: 2 (1,2) Reader 2: 2 (1,2)	Reader 1: 2 (1,2) Reader 2: 2 (1,2)
125ms	Reader 1: 2 (2,3) Reader 2: 2 (2,3)	Reader 1: 3 (2,3) Reader 2: 3 (2,3)	Reader 1: 3 (2,3) Reader 2: 3 (2,3)

*Abbreviations* AVC, aortic valve calcifications; CAC, coronary artery calcium; MAC, mitral annular calcification

Supplementary Fig. 1 illustrates that in 66ms reconstructions, motion artifacts were not only reduced in patients with heart rates above 80 beats per minute but also in patients with heart rates below 70 beats per minute compared to 125ms reconstructions.

## Discussion

This study investigated the impact of temporal resolution on CAC, AVC, and MAC scores in patients undergoing non-contrast cardiac CT. Our findings indicated that - although the agreement and correlation of calcium scores quantified on 66 ms and 125 ms reconstructions were high - calcium scores of CAC, AVC, and MAC were significantly higher on 125 ms reconstructions. Subsequently, CAC risk categories were overestimated in 4% and likelihood categories for severe aortic stenosis were overestimated in 11% of patients. Subjective analysis revealed that motion artifacts and blurring of calcifications were significantly more frequent on 125 ms reconstructions as compared with 66 ms reconstructions.

In this study we employed a recently introduced first-generation dual-source PCD-CT for non-contrast cardiac imaging. This system can outperform previous EID-CT systems for cardiac imaging as its dual-source mode can achieve multienergy imaging at the highest temporal resolution of 66 ms with pitch values of up to 3.2 [21]. In contrast, previous high-end dual-source EID-CT systems also allow for cardiac imaging at a temporal resolution of 66 ms, but without the possibility of multienergy imaging and with restrictions towards the pitch values. Nonetheless, this shows that this study could also have been performed on a latest generation dual-source EID-CT system and that our findings are thus also applicable to other scanners.

Coronary arteries undergo significant variations of motion velocities during the cardiac cycle depending on the coronary artery segment and the heart rate. The RCA exhibits the highest mean velocity (of up to 38 mm/s) during the cardiac cycle [18]. If the motion velocity of a coronary artery exceeds the temporal resolution of the non-contrast cardiac CT scan, motion artifacts occur and, as a result, calcified plaques may appear blurred.

Early investigations performed on 64-slice multidetector CT, having a temporal resolution of 165ms, indicated an impact of cardiac motion and heart rate on calcification quantification [16, 17]. More recently, Van der Werf et al. revisited this topic and evaluated the influence of heart rate on coronary calcium scores using a dynamic phantom on different state-of-the art EID-CT with temporal resolutions ranging between 175 ms and 75 ms [19]. They found that calcium scores of medium and high mass calcifications significantly increased by up to 50% at heart rates above 75 beat per minute irrespective of the scanner.

Our study corroborates these findings as median CAC scores as well as AVC and MAC scores were higher on 125 ms than on 66 ms reconstructions. Notably, this overestimation resulted in the reclassification of calcium scoring categories in some patients, whereby the cardiovascular risk and the likelihood for the presence of severe aortic stenosis were overestimated on 125 ms reconstructions as opposed to 66 ms reconstructions. This can potentially influence the treatment of patients for example with additional cardiovascular diagnostics or different therapeutic strategies [27].

In this regard, Van der Werf et al. hypothesized that the higher CAC score may be due to motion blur of the calcifications increasing the number of voxels above the threshold of 130 HU [19]. Accordingly, our subjective analysis showed significantly worse image quality and more motion artifacts for CAC, AVC, and MAC on 125 ms than on 66 ms reconstructions.

Mergen et al. recently showed that ultra-high-resolution coronary CT angiography with dual-source PCD-CT provided less motion artifacts and superior image quality at a temporal resolution of 66 ms as compared with 125 ms. Authors did not observe a significant correlation between vessel sharpness and average heart rate or heart rate variability. However, only a small sample size was included in their study potentially hampering a meaningful statistical evaluation [20]. In the study presented here, a significant impact of average heart rate on the absolute difference in calcium score between both reconstructions was found only for the LAD. No significant impact of heart rate variability on the absolute difference in calcium scores between both reconstructions was found. Leschka et al. found a significant correlation between heart rate variability and overall image quality of coronary CT angiography using a single-source EID-CT with a temporal resolution of about 185 ms. This result may potentially influenced by the lower temporal resolution of that scanner compared to both the dual-source and single-source image reconstructions with the PCD-CT used in our study.

It is important to note that the process of calcium quantification is susceptible to a variety of confounding technical and non-technical factors such as the CT scanner, reconstruction methods, slice thickness, image noise, cardiac motion, reader variability, and others [8, 11, 12, 28]. To introduce some level of standardization, McCollough et al. introduced a standardized protocol for CAC scoring in 2007 [14]. Our study adds to the literature that temporal resolution of CT scanners is an additional and important parameter influencing the quantification of cardiac calcium.

The here presented investigation had the following limitations: First, this was a single center study with a limited number of subjects who underwent imaging on a single scanner from a single vendor. Thus, further studies corroborating our findings are warranted. Second, the impact of only two temporal resolutions on calcifications was investigated. Third, non-contrast cardiac scans were acquired in the ECG-gated sequential mode at an absolute interval of 280 ms from the R-wave. The intra-individual impact of a reduced temporal resolution on calcium scores using different acquisition modes (e.g., high-pitch mode) or different trigger phases remains to be determined. Finally, no outcome data was available and the consequences of the reclassifications of patients remain unclear.

In conclusion, temporal resolution has a significant impact on calcium scoring with cardiac CT, with CAC, MAC, and AVC being overestimated on lower temporal resolution images likely due to increased image blurring eventually leading to an overestimation of the patients risk.

## Electronic supplementary material

Below is the link to the electronic supplementary material.

Supplementary Material 1

Supplementary Material 2

Supplementary Material 3

## Abbreviations

AVC: Aortic Valve Calcium
CAC: Coronary Artery Calcium
CAD: Coronary Artery Disease
CX: Circumflex Artery
ECG: Electrocardiography
EID: Energy Integrating Detector
ICC: Intraclass Correlation Coefficient
LAD: Left Anterior Descending Artery
LM: Left Main Coronary Artery
MAC: Mitral Annular Calcium
PCD: Photon Counting Detector
QIR: Quantum Iterative Reconstruction
RCA: Right Coronary Artery
ROI: Region of Interest
SNR: Signal to Noise Ratio
TAVR: Transcatheter Aortic Valve Replacement
