# Supplementary material for: Effect of temporal resolution on calcium scoring: insights from photon-counting detector CT
Source: Int J Cardiovasc Imaging. 2024 Feb 23;41(3):615–25. doi: 10.1007/s10554-024-03070-6 (PMC11880162; doi:10.1007/s10554-024-03070-6)
Supplement: Supplementary file 1 — Supplementary Material 1 [file 10554_2024_3070_MOESM1_ESM.docx]

**Supplementary Material**

**Table 1S:** Overview of coronary artery calcium (CAC), aortic valve calcium (AVC), and mitral annular calcium (MAC) mass and volume scores stratified by temporal resolution. Scores are provided as median (interquartile range). P-values for groupwise comparisons (66 ms vs 125 ms temporal resolution) are shown.

Abbreviations: Cx, Circumflex coronary artery; LAD, left anterior descending; LM, left main coronary artery; RCA, right coronary artery.

| ***Mass*** | **66 ms** | **125 ms** | **p-value** | ***Volume*** | ***66 ms*** | **125 ms** | **p-value** |
| --- | --- | --- | --- | --- | --- | --- | --- |
| **Overall CAC** | 61.3 (28, 132.1) | 63.1 (34.6, 135.4) | **<0.001** | **Overall CAC** | 442 (236.1, 880.7) | 476.8 (203.9, 923) | <0.001 |
| **LM CAC** | 0.2 (0, 7.8) | 0.3 (0, 7.5) | **<0.01** | **LM CAC** | 2.6 (0, 56.1) | 3.8 (0, 59.3) | **<0.001** |
| **LAD CAC** | 20 (7.9, 52) | 21.1 (8.6, 56) | **<0.001** | **LAD CAC** | 153.2 (58.1, 351.6) | 161.4 (60.5, 357.4) | **<0.001** |
| **CX CAC** | 7.7 (0.9, 24.7) | 8 (0.9, 24.8) | **<0.01** | **CX CAC** | 57 (8.6, 198) | 61.3 (7.9, 203.9) | <0.01 |
| **RCA CAC** | 15.4 (1.4, 48) | 14.5 (1, 48.1) | **<0.01** | **RCA CAC** | 124.4 (12.3, 370) | 129.4 (9.2, 382) | **<0.001** |
| **AVC** | 416.2 (289.3, 642.4) | 437.2 (295.5, 660.7) | **<0.001** | **AVC** | 2219.1 (1616, 3102.6) | 2480.5 (1713.9, 3342.2) | **<0.001** |
| **MAC** | 25.4 (0, 217.4) | 30.5 (0, 227.1) | **<0.001** | **MAC** | 214.2 (0, 1067.7) | 222.8 (0, 1263) | **<0.001** |
