# Supplementary figures and images for: Effect of temporal resolution on calcium scoring: insights from photon-counting detector CT
Source: Int J Cardiovasc Imaging. 2024 Feb 23;41(3):615–25. doi: 10.1007/s10554-024-03070-6 (PMC11880162; doi:10.1007/s10554-024-03070-6)

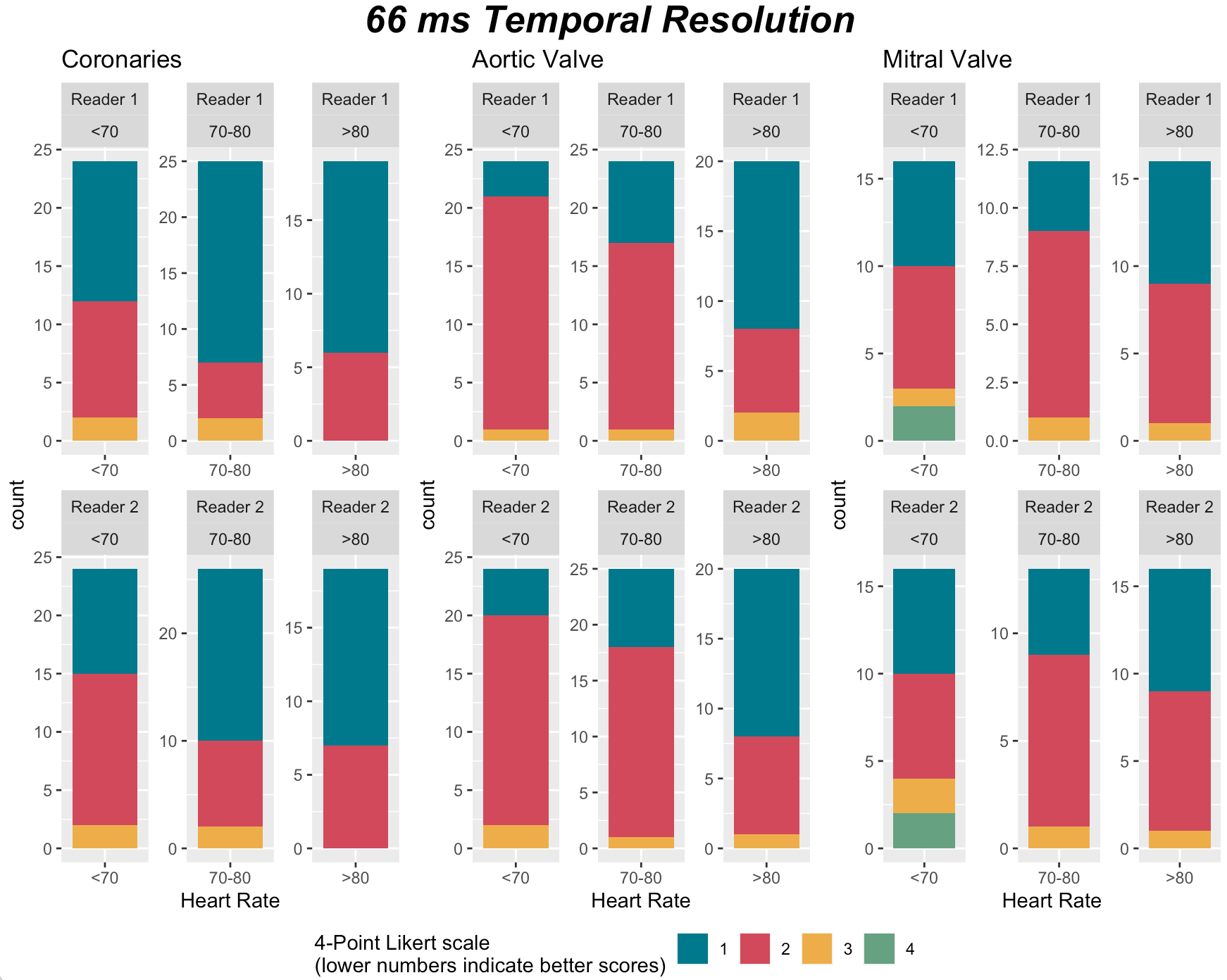

Supplement: Supplementary file 2 — Supplementary Material 2 [file 10554_2024_3070_MOESM2_ESM.tif]

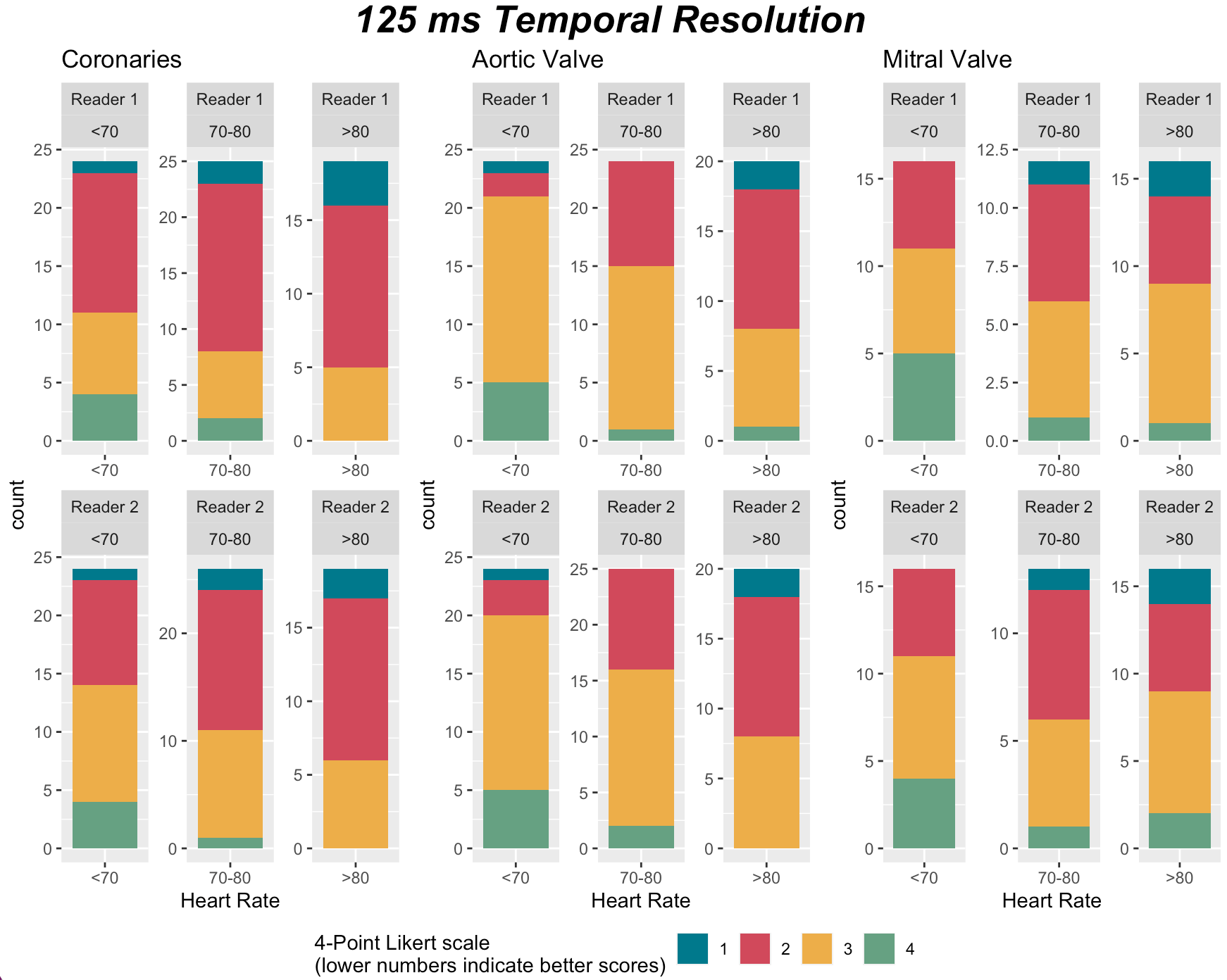

Supplement: Supplementary file 3 — Supplementary Material 3 [file 10554_2024_3070_MOESM3_ESM.tif]
